# Angioplasty of the inferior vena cava with a bovine pericardial patch by the modified open-chest dorsal approach for Budd-Chiari syndrome: A case report

**DOI:** 10.1016/j.ijscr.2023.108946

**Published:** 2023-10-10

**Authors:** Akihiko Soyama, Shinichiro Ogawa, Takanobu Hara, Tomohiko Adachi, Takashi Miura, Susumu Eguchi

**Affiliations:** a Department of Surgery, Nagasaki University Graduate School of Biomedical Sciences, Nagasaki, Japan; b Department of Cardiovascular Surgery, Nagasaki University Graduate School of Biomedical Sciences, Nagasaki, Japan

**Keywords:** Case report, Bovine pericardial patch, Endovenectomy, Liver transplantation

## Abstract

**Introduction and importance:**

Surgical treatment of Budd-Chiari syndrome (BCS) includes endovenectomy followed by angioplasty of the inferior vena cava (IVC). Herein, we report a case of surgery using an open-chest approach in a patient with BCS. We modified the technique reported by Kuniyoshi et al.

**Case presentation:**

A 45-year-old male, was diagnosed with BCS and referred to our hospital. We used an open-chest approach to remove stenosis in the IVC and angioplasty with a bovine pericardial patch. Endovenectomy and angioplasty were performed by clamping the stenosis above and below it with Pringle's clamping under extracorporeal circulation. The patient is currently undergoing outpatient follow-up 14 months after the surgery, and his liver function and blood test results were normal, with no symptoms.

**Clinical discussion:**

The main advantage of this technique is that the liver is not mobilized from the diaphragm, which allows for the preservation of collateral blood flow between the diaphragm and liver, reducing the amount of intraoperative blood loss and damage to the liver parenchyma due to intraoperative congestion. In addition, no mobilization of the liver from the diaphragm will prevent future surgical difficulties due to adhesions during total hepatectomy when liver transplantation becomes necessary.

**Conclusion:**

The techniques described in this article include procedures that cardiovascular surgeons usually perform such as thoracotomy, pericardiotomy, and extracorporeal circulation. Collaborative work by hepatobiliary surgeons and cardiovascular surgeons can achieve successful outcomes with this procedure in patients with BCS.

## Highlights

• Surgical treatment for Budd-Chiari syndrome includes endovenectomy followed by angioplasty.
• Kuniyoshi, et al. reported approach from the thorax to the dorsal side of IVC.
• We modified the surgery reported by Kuniyoshi, et al. with Pringle maneuver.
• Hepatobiliary surgeons should be familiar with these techniques.

## 1 Introduction

Surgical treatment for Budd-Chiari syndrome (BCS) includes endovenectomy followed by angioplasty of the inferior vena cava (IVC) [1,2]. In addition, liver transplantation may be indicated when the lesion is accompanied by liver failure or symptoms of portal hypertension [1,3]. Because the thoracic IVC needs to be approached, surgery for BCS has often been reported in the field of thoracic and cardiovascular surgery. Kuniyoshi et al. reported a technique in which the liver is approached from the thorax to the dorsal side of the IVC without mobilizing the liver from the diaphragm, followed by resection of the stenotic lesion of the IVC, open endovenectomy, and angioplasty of the IVC [4]. This method can be implemented while preserving drainage from the liver via the diaphragm, helping to compensate for the reduced flow of the hepatic vein and preventing complications such as bleeding and parenchymal injury associated with the mobilization of the liver.

Furthermore, if liver transplantation is necessary in the future, this approach can help reduce the difficulty of liver transplantation, including bleeding during the dissection phase of total hepatectomy associated with adhesion between the liver and the diaphragm. Given the above, the method reported by Kuniyoshi et al. is considered useful for helping hepatobiliary surgeons who perform BCS for the possibility of liver transplantation [5].

Herein, we report angioplasty of the IVC with a bovine pericardial patch via the open chest dorsal approach for BCS.

## 2 Case report

Ethical approval for this case report was provided by the Institutional Review Board of Nagasaki University Hospital (No. 19102143) on October 22, 2019.

This work was reported in line with SCARE criteria [6].

**Fig. 1:**
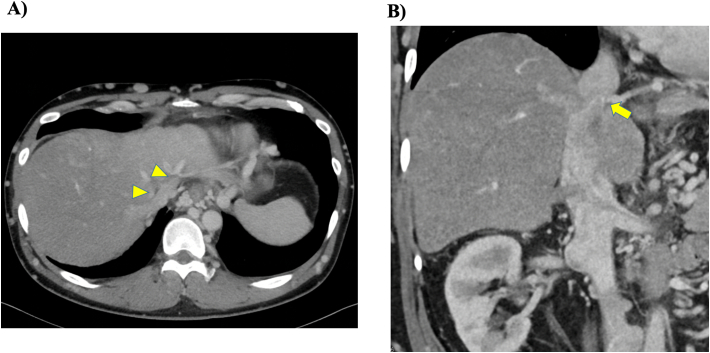
Preoperative contrast-enhanced computed tomography. Stenosis of the IVC just above the confluence of hepatic veins. Heterogenous enhancement of parenchyma. Development of esophageal varices. Allow shows the stenotic lesion of the IVC. Allow heads show the occluded middle hepatic vein and left hepatic vein.

**Fig. 2:**
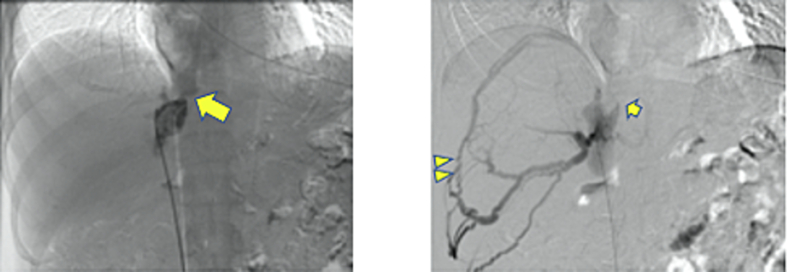
Preoperative venography. Collateral vessels connected from the peripheral branch of the right hepatic vein into the diaphragmatic vein (Allows). The arrow shows the stenotic lesion of the IVC.

A 45-year-old male, was diagnosed with BCS and referred to our hospital. Contrast-enhanced computed tomography (CT) showed stenosis of the IVC just above the confluence of the hepatic veins (Fig. 1), and angiography of the IVC showed membranous stenosis and collateral vessels connected from the peripheral branch of the right hepatic vein into the diaphragmatic vein (Fig. 2). There was evidence of portal hypertension with decreased platelet count and esophageal varices. There were no ascites or encephalopathy, and his albumin level was maintained at 4.2 g/dl, but the total bilirubin level was elevated at 2.4 g/dl, and prothrombin time was slightly decreased to 66 %.

The patient's chief complaint was an impaired quality of life due to marked edema in the lower body. The possibility of stenting the stenotic lesion of the IVC by interventional radiology was considered, but a direct approach to the IVC and reopening of the occluded IVC and hepatic veins under cardiopulmonary bypass was ultimately decided as the approach to resolve not only the stenosis of the IVC but also the obstruction of the middle and left hepatic veins. The patient underwent a direct approach to the IVC and reopening of the occluded IVC and hepatic veins under cardiopulmonary bypass to treat the IVC stenosis and hepatic vein occlusion. We planned an open-chest approach to remove the stenosis in the IVC and reopen the occluded hepatic veins and angioplasty with a bovine pericardial patch by collaborative surgery with a cardiovascular surgeon by clamping the inferior vena cava above and below the stenosis with Pringle's clamping under extracorporeal circulation.

## 3 Operative procedure (Fig. 3, Video S1)

The patient was placed in the left semi-prone position. The right femoral artery and vein were taped in preparation for the extracorporeal circulation. An incision was made continuously from the sixth intercostal space above the umbilicus, with the right lateral border at the axillary line and the left lateral border at the midline. During laparotomy, the umbilical ligament was preserved as much as possible to preserve the collateral blood vessels.

**Fig. 3:**
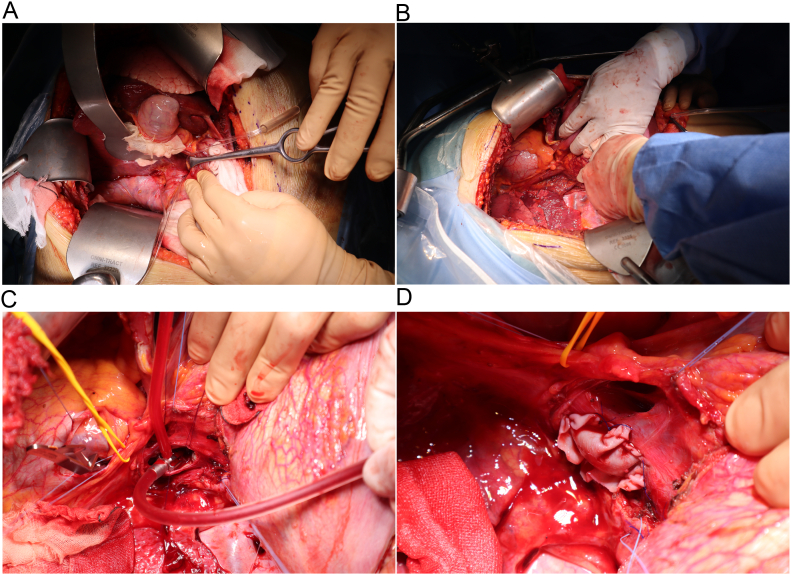
Operative procedures. A) Encircling the hepatoduodenal ligament and infrahepatic inferior vena cava. B) Exposing the supradiaphragmatic inferior vena cava. C) Endovenectomy of the inferior vena cava with division of the diaphragm. Yellow tape encircles the right phrenic nerve. D) Angioplasty with a bovine pericardial patch. (For interpretation of the references to colour in this figure legend, the reader is referred to the web version of this article.)

The diaphragm was incised caudally on the right side of the liver under one-lung ventilation. The collateral vessels between the diaphragm and the liver were preserved. The hepatoduodenal ligament was encircled in preparation for the subsequent Pringle procedure (Fig. 3A). Taping of the infrahepatic IVC was performed. The IVC was markedly dilated. Surrounding tissues were carefully dissected and taped (Fig. 3A).

To rotate the liver to the left, we proceeded to partially incise the diaphragm on the cranial side of the right lobe of the liver and confirmed the location of the IVC (Fig. 3B). During this procedure, the diaphragm was incised in a line to preserve the right inferior diaphragmatic vein. This maneuver allowed the liver to be adequately rotated to the left and the dorsal aspect of the IVC was safely identified just above the diaphragm. The surrounding tissues were then dissected.

After encircling and securing the right phrenic nerve, we encircled the IVC in the pericardial sac in preparation for the subsequent incision of the IVC, removal of the membranous part, and patch plasty. The dorsal surface of the IVC was gradually revealed by incising the diaphragm on the dorsal surface with an energy device (LigaSure™; Medtronic, Dublin, Ireland) After palpation and transesophageal ultrasound (US) revealed an area of stenosis, we proceeded with the diaphragmatic incision from the cranial side towards the caudal side at the 6 o'clock point on the IVC so that sufficient working space could be secured. As further diaphragmatic incision might cause damage to the collateral vessels, the incision was limited to the same area. The stenosis was again checked, it was solidly palpated, and the same area was confirmed by transesophageal US. We decided to incise the IVC as planned, and endovenectomy was performed after approaching the stenotic site (Fig. 3C). Subsequently, angioplasty of the IVC was performed using a bovine pericardial patch (Fig. 3D).

After setting the preparation for extracorporeal circulation, the infrahepatic and intrapericardial IVC were clamped, and an incision was made in the IVC using the Pringle maneuver. Observation of the inside of the IVC revealed a white, organizing membranous structure that was considered the cause of the stenosis. While removing the membrane-like structure, the suction tube was inserted without resistance to the caudal side of the IVC. Further removal of the remaining membranous structures allowed the insertion of the suction tube without resistance into the left hepatic vein. At this point, we determined that the stenosis caused by the membranous structures had been removed. The incision was 4 cm in length and a bovine pericardial patch (Edwards Lifesciences, Irvine, CA, USA). IVC patch plasty was performed with continuous 4-0 monofilament non-absorbable sutures. When IVC angioplasty was completed, the clamp was released. From IVC incision to the patch-forming procedure, the total ischemic time of the liver using the Pringle method was 35 min. The pericardium was closed using continuous suturing. A 24-Fr thoracic tube was inserted into the thoracic cavity through the eighth intercostal space. Blood pressure and other vital signs were stable during the surgical procedures. The operation time was 6 h 53 min and the estimated blood loss was 641 g.

**Fig. 4:**
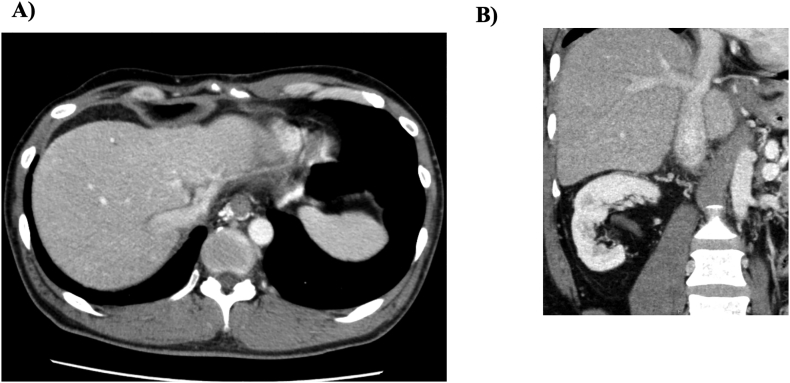
Postoperative contrast-enhanced computed tomography. A) Wide orifice of the right and left hepatic vein. Homogenous enhancement of the liver parenchyma. B) Improvement of the stenotic lesion in the IVC.

The patient was extubated on the first postoperative day and was discharged from the intensive care unit on the second day. Postoperatively, the patient developed cardiac tamponade due to bleeding in the pericardial sac and underwent pericardial drainage; however, his condition stabilized thereafter, and he was discharged home. Postoperative contrast-enhanced CT on postoperative day 9 showed a widely patent IVC and orifice of the hepatic veins, with homogenous enhancement of the liver parenchyma (Fig. 4). Furthermore, both esophageal varices and collateral vessels in the abdominal wall were markedly diminished (Fig. 4). The postoperative hospital stay was 44 days. The patient is currently undergoing outpatient follow-up at 14 months after the surgery, and his liver function and blood test results were normal, with no preoperative symptoms.

## 4 Discussion

The procedure described herein for BCS, in which an open thoracic approach was performed at the dorsal side of the IVC from the thoracic cavity, was performed safely. With this procedure, as in the case presented here, the imaging findings and symptoms improved rapidly after surgery.

The original technique of this procedure reported by the University of the Ryukyus group approaches the lesion directly via a right thoracoabdominal incision and is appropriate for repairing lesions of both the IVC and hepatic veins simultaneously [7]. Inafuku et al. reported their experience performing open endvenedectomy with a pericardial patch graft for correction of BCS using an open-chest dorsal approach in 53 consecutive patients. The 5- and 10-year patency rates without reoperation or PTV for reconstructed IVC were 90.5 % and 84.3 %, respectively [2]. The postoperative hospital stay was 49 (range, 15–124) days. Furthermore, the same group reported that the blood loss during the operation was 1165 ± 1000 cc in 69 patients who underwent this procedure.

The main advantage of this technique is the approach to the IVC without mobilization of the liver, which allows for the preservation of collateral blood flow between the diaphragm and liver, thereby reducing the amount of intraoperative blood loss and reducing damage to the liver parenchyma due to intraoperative congestion.

In addition, no mobilization of the liver from the diaphragm will prevent future surgical difficulties due to adhesions during total hepatectomy when liver transplantation becomes necessary. In particular, in living donor liver transplantation, the recipient's native IVC may have to be preserved [3]; if the IVC is completely exposed during the initial surgery prior to liver transplantation and IVC angioplasty is performed, preservation of the IVC and subsequent reconstruction may be extremely difficult. However, there have been no reports of cases in which liver transplantation was performed after this method was used, probably because of the excellent outcome of this method, showing a good vascular patency rate. The impact of the method reported here on future surgical treatments for BCS, including liver transplantation, needs to be evaluated based on the accumulation of more cases in the future.

A previous report described excellent results with IVC incision, endovenectomy, and subsequent angioplasty, without interrupting blood flow. However, in the method reported in this study, we performed the Pringle procedure. TVE was achieved during liver surgery by clamping the suprahepatic and infrahepatic IVC and performing the Pringle procedure, total vascular occlusion (TVE) during liver surgery was achieved. Favorable outcomes have been reported in patients undergoing hepatic resection with TVE have been reported [8,9]. Using the Pringle maneuver as part of TVE allows for a better view of the inside of the IVC during the procedure. We believe that including a hepatobiliary surgeon skilled in liver handling and postoperative management as a member of the surgical team for BCS can aid in performing the procedure described here.

In the present case, we used a bovine pericardial patch for angioplasty of the IVC, and good results with this technique have been reported [10]. Although long-term follow-up is important in this case, the bovine pericardial patch is a useful tool for IVC reconstruction.

## 5 Conclusion

Although the techniques described in this article include procedures that cardiovascular surgeons usually perform, such as thoracotomy, pericardiotomy, and extracorporeal circulation, hepatobiliary surgeons also familiarize themselves with these techniques in preparation for handling the liver in BCS and for future transplantation. This method is also expected to be useful as an approach to IVC of the liver. Collaborative work by hepatobiliary surgeons and cardiovascular surgeons can achieve successful outcomes with this procedure in patients with BCS.

The following is the supplementary data related to this article.

Supporting video 1 Radical open endvenectomy was followed by angioplasty of the inferior vena cava with a bovine pericardial patch using the open chest dorsal approach. Supporting video 1

## Consent

Written informed consent was obtained from the patient for publication and for any accompanying images. A copy of the written consent is available for review by the Editor-in-Chief of this journal upon request.

## Ethical approval

This study was approved by the institutional review board, and informed consent was obtained from the patient. Ethical approval for this case report was provided by the Institutional Review Board of Nagasaki University Hospital (No. 19102143) on October 22, 2019.

## Funding

This case report received no specific grants from any funding agency in the public, commercial, or not-for-profit sectors.

## Author contribution

(I) Conception and design: Soyama, Eguchi.
(II) Administrative support: Ogawa, Hara, Adachi.
(III) Provision of study materials or patients: Soyama, Ogawa, Hara.
(IV) Collection and assembly of data: Soyama, Ogawa, Hara.
(V) Data analysis and interpretation: Soyama, Ogawa, Hara, Adachi, Miura, Eguchi.
(VI) Manuscript writing: Soyama, Eguchi.
(VII) Final approval of manuscript: All of the authors.

## Guarantor

The guarantor is Akihiko Soyama.

## Research registration number

This report is not applicable to this topic.

## Conflict of interest statement

The authors declare that they have no conflicts of interests.

## Data Availability

The data supporting the findings of this study are available from the corresponding author upon reasonable request.
